# Characterization of Plant-Based Raw Materials Used in Meat Analog Manufacture

**DOI:** 10.3390/foods14030483

**Published:** 2025-02-03

**Authors:** Viorica Bulgaru, Mihail Mazur, Natalia Netreba, Sergiu Paiu, Veronica Dragancea, Angela Gurev, Rodica Sturza, İlkay Şensoy, Aliona Ghendov-Mosanu

**Affiliations:** 1 Faculty of Food Technology, Technical University of Moldova, 9/9 Studentilor St., MD-2045 Chisinau, Moldova; mihail.mazur@saiem.utm.md (M.M.); natalia.netreba@tpa.utm.md (N.N.); sergiu.paiu@doctorat.utm.md (S.P.); veronica.dragancea@chim.utm.md (V.D.); angela.gurev@chim.utm.md (A.G.); rodica.sturza@chim.utm.md (R.S.); aliona.mosanu@tpa.utm.md (A.G.-M.); 2 Department of Food Engineering, Middle East Technical University, 06800 Ankara, Turkey; isensoy@metu.edu.tr

**Keywords:** plant-based materials, protein, meat analogs, in vitro digestibility, antioxidant activity

## Abstract

The purpose of this research was to investigate the characteristics of different plant-based sources rich in protein, chickpea flour (CPF), hazelnut oil cake (HOC), soy protein isolate (SPI) and concentrate (SPC), and pea protein isolate (PPI) for their subsequent use in the manufacture of meat analogs. The protein sources were analyzed for dry matter, ash, protein, fat, starch, dietary fiber, water holding capacity, granulosity, color parameters (L*, a*, b*, C*, YI), antioxidant activity before and after gastrointestinal in vitro digestion, and amino acid and mineral compositions. The highest dry matter content was determined in hazelnut oil cake and pea protein isolate. For the protein content, maximum values were obtained for the protein isolate and concentrate samples, from 52.80% to 80.50%, followed by hazelnut oil cake and chickpea flour. The water-holding capacity of all plant sources was directly influenced by the values of protein content, dietary fiber, and granulosity. The results obtained after gastrointestinal digestion also showed quite significant antioxidant activity, which is due to the process of hydrolysis and denaturation of plant-based protein sources in the gastrointestinal tract. Major amino acids identified in the analyzed samples were glutamic acid, leucine, arginine, phenylalanine, serine, valine, alanine, and tyrosine from minerals P, Na, Mg, and Ca. Principal component analysis (PCA) was used to illustrate the relationship between physicochemical characteristics, amino acid composition, mineral composition, and antioxidant activity determined in the plant-based materials.

## 1. Introduction

The idea of developing a range of food products as alternatives to meat has been known since ancient times, starting with the development of tofu as a source of protein by the Chinese, later gaining popularity in Japan [1]. Later, in the early 20th century, nut- and seed-based products were developed to promote human health [2]. During this period, in industrialized countries, interest in developing the range of textured vegetable proteins from cereals, legumes, or nuts as raw materials increased in the context of agriculture expansion [3].

Today, meat analogs are in greater demand due to several influencing factors:

- The impact of raising animals on the environment, which requires 70% of agricultural land for their needs [4], increasing greenhouse gas emissions [5];
- Increasing the number of vegetarians, taking into account religious beliefs and eating habits [6];
- Food security in light of the increased price of meat compared to plant-based raw materials [5];
- Public health regarding the consumption of red meat is associated with the occurrence of various diseases (cardiovascular, cancer, obesity) [7,8].

Consumer acceptance of meat analogs still faces some challenges related to flavor, texture, and appearance [9]. A comparing study of the quality of meat and meat analogs showed that besides the fact that classic pork, beef, and poultry meat were quantitatively richer in protein by about two times, plant-based meat analogs contained significant amounts of carbohydrates and dietary fiber. These nutrients were not found in meat. In addition, meat analogs were rich in sodium (eight times higher) and polyunsaturated fatty acids and had a low cholesterol content. Also, the meat analogs were noted for their lower protein digestibility [10].

The specific quality characteristics of meat analogs are determined by the ingredients used in their manufacturing recipes, such as plant-based proteins responsible for creating the fibrous structure (texture), which is considered by researchers to be a major problem in the formation of meat analogs [11,12], oil, pigments, and binding agents necessary for the formation of the shape, color, and taste of the finished product [13,14], as well as the technological processes used, such as electrospinning, 3D printing, shearing, and extrusion [15]. Extrusion technology is classified into low moisture extrusion cooking (10–40% (*w*/*w*) water) and high moisture extrusion cooking (up to 70% (*w*/*w*) water) [16].

The correct choice of protein-rich raw materials is responsible for solving the problems of obtaining an appropriate fibrous structure for meat analogs, which is as close as possible to that of meat. The degree of using vegetable protein sources in manufacturing meat analogs depends on their availability, price, and technological properties [17]. Plants positively influence the process of biodiversity conservation, agriculture, and soil fertility preservation [3]. Various legumes (20–35% protein content) [18,19], nuts (39.4–42.1% protein content) [20], oilseeds (about 45% proteins) [4,19], and microalgae with an average of over 50% protein content [21] could be an important source of plant-based protein in manufacturing meat analogs if they meet the respective requirements [22]. The technological functions (solubility, thermal stability, emulsification, flavor binding capacity, and digestibility) of proteins are most relevant to the creation of meat analogs, as they are manifested through their use as a formulation, concentrate, or isolate [23]. Of all the types of proteins contained in plant sources, albumin is of particular importance because it can form complex bonds with carbohydrates, lipids, and nucleic acids [24].

The purpose of this research work was to characterize the quality indices of plant-based raw materials (chickpea flour (CPF), hazelnut oil cake (HOC), pea protein isolate (PPI), soy protein concentrate (SPC), and soy protein isolate (SPI)), with potential for use in the manufacture of meat analogs.

## 2. Materials and Methods

### 2.1. Chemicals

n-hexane (>95%), hydrogen peroxide (30%), hydrochloric acid (38%), potassium bicarbonate (97%), nitric acid ASC reagent (70%), perchloric acid ASC reagent (70%), deionized water, ninhydrin, pepsin (extra pure, powder), tripsin (lyophilized powder), and sodium bicarbonate were provided by Sigma-Aldrich (Schnelldorf, Germania). Petroleum ether hydroxide, acetone, and n-octan were purchased from Chemapol (Prague, Czech Republic). Kjeldahl mineralization Catalyst CX 5.5 GR was purchased from VWR Chemicals (Lutterworth, UK). Vreceiver TKN (Bromocresol green and Methyl red) was obtained from Velp Scientifica (Usmate, Italia). Gallic acid (97%), sodium hydroxide (≥97%), phenolphthalein, 2,2-diphenyl-1-picrylhydrazyl-hydrate (DPPH) (≥95%), and ABTS (2,2-azino-bis(3-ethylbenzothiazoline-6-sulphonic acid) (ABTS) (≥98%) were purchased from Merck (Darmstadt, Germany). A Specord 200 Plus spectrophotometer (Jena, Germany) was used for spectrophotometric measurements.

### 2.2. Raw Material

Chickpea, a variety of Ichel, was received from the Institute of Genetics, Physiology and Plant Protection, located in Chisinau, Republic of Moldova.

Chickpea grains were ground in a 3100-laboratory mill (Perten Instruments, Hägersten, Sweden).

**Figure 1:**
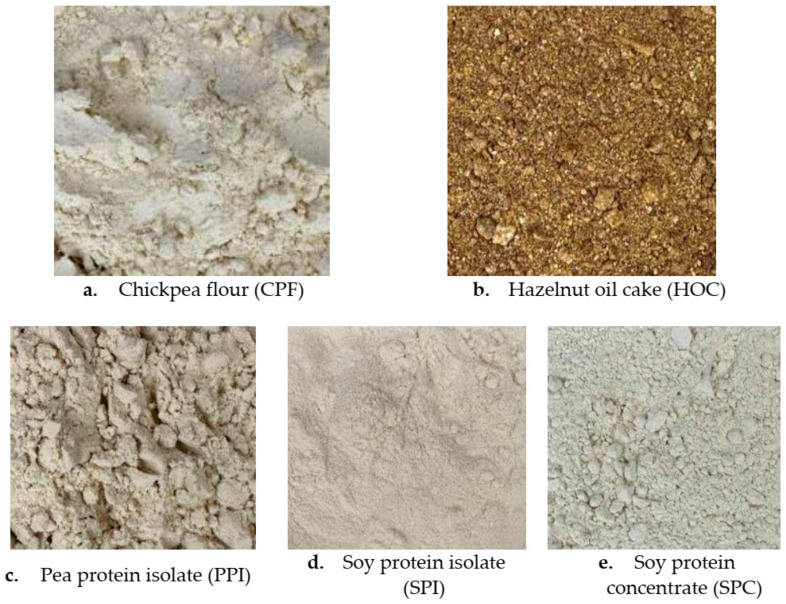
Plant-based materials.

HOC (Altaş Yağ Sanayi Ticaret A.Ş., Ordu, Turkey), along with SPC, SPI, and PPI (Kimbiotek Kimyevi Maddeler San. Tic. A.Ş., Istanbul, Turkey), were received from the Department of Food Engineering, Middle East Technical University, Ankara, Türkiye. All samples are presented in Figure 1.

### 2.3. Physicochemical Quality Parameters

The dry matter (DM) was determined by the oven-drying method, which measures the weight loss due to the evaporation of water (AOAC Official Method 925.10). The three parallel samples of analyzed material were weighed to two decimal places using an analytical balance and dried in a drying oven at 103 °C to constant weight (>4 h). After drying, the sealed samples were placed in a desiccator and weighed after cooling. DM (%) was calculated according to Equation (1):

(1) $$DM=100-\left(\frac{m_{1}-m_{2}}{m_{0}}\times 100\right),$$

where

*m*_0_—mass of the sample weight for drying, g;
*m*_1_—mass of the sample and the aluminum moisture box before drying, g;
*m*_2_—mass of the sample and the aluminum moisture box after drying, g.

Ash content (AC) represents the mineral content determined using the dry ashing method, which measures the inorganic residue remaining after combustion by burning the organic material in a muffle furnace at 550 °C, according to AOAC (2006). After cooling in a desiccator, the total mass of the crucible and the ash was measured in two decimal places. AC results for analyzed ash samples were expressed as a percentage of the initial sample mass.

The protein content (PC), fat content (FC), and crude fiber content (CFC) of the samples were determined using the methods reported by the AOAC (2012).

PC was determined according to the Kjeldahl Method in a UDK129 (VELP Scientifica, Italy) [25] by measuring the nitrogen content of the sample and converting it to protein using a conversion factor (typically 5.70 for cereal and legumes; 5.46 for HOC).

FC was determined in a SER148 Solvent Extraction Unit (VELP Scientifica, Monza, Italy) [26] by the Soxhlet extraction method using hexane and measuring the mass of the extracted fat (AOAC Official Method 948.22). After the 4 h extraction process, the extracted fat was separated, and the oil/fat-containing flasks were connected to a rotary evaporator to remove most of the solvent. The flasks were placed in a dry oven at 103 °C for 30 min to evaporate any remaining solvent, then dried, cooled in a desiccator, and reweighed.

The procedure for measuring CFC in FIWE Raw Fiber Extractors (VELP Scientifica, Italy) includes a series of chemical treatments designed to eliminate all soluble and easily digestible substances from the sample. The remaining fiber components were rinsed, dried, and weighed to assess the residual fiber content. The CFC was expressed as a percentage of the original mass of the analyzed sample.

The starch yield (*SY*) was determined using the wet milling method, following the procedure outlined by da Silva et al. [27], with adjustments as described by Santos et al. [28]. The *SY* (%) was calculated using Equation (2):

(2) $$SY=\frac{DSM}{RMM}\times 100$$

where

*DSM*—mass of the dry starch, g;
*RMM*—mass of the raw material, g.

### 2.4. Amino Acid Profile 

The amino acid profile was determined according to the standard protocols established by the Association of Official Analytical Chemists (AOAC). Specifically, the analysis involved cation-exchange chromatography, a technique separating amino acids based on their charge. Following separation, the amino acids were subjected to post-column derivatization with ninhydrin, a reagent that reacts with amino acids to produce a measurable colored product. The resulting color intensity was measured using a spectrophotometer, allowing for precise quantification of individual amino acids. Quantification of the amino acids was then carried out by AOAC Official Method 982, ensuring accurate and standardized results [25]. The AOAC method ensures high accuracy, reproducibility, and consistency, making it the standard choice for regulatory and nutritional labeling purposes. Technical details: The tubes for columns of resin OSTION IG ANB 5–10 µm, 22 cm in height. Photometer with a wavelength of 570 nm. Flow rate 12 mL per hour. Mobile phase composition: citric acid, lithium citrate, lithium chloride, 2N LiOH, HCl conc., thiodiglycol, and ethyl alcohol. Buffer solutions (pH 2.9, pH 2.95, pH 3.2, pH 3.8, pH 5.0). Standard used SIGMA AA-S-18.

### 2.5. Mineral Profile

The mineral composition of the samples was analyzed following a standardized wet digestion procedure, which ensured the precise breakdown of organic matter and release of inorganic elements for subsequent quantification [29]. A total of 0.5 g of the analyzed sample was accurately weighed and processed using a mixture of 5 mL of concentrated nitric acid (HNO_3_) and 1 mL of concentrated perchloric acid (HClO_4_). This combination of acids effectively oxidizes organic material and dissolves the mineral components, converting them into a solution suitable for analytical measurement.

The resulting solution was filtered to remove any insoluble residues, ensuring a clear, particle-free sample. The filtrate was then diluted with deionized water to a final volume of 100 mL in a calibrated volumetric flask, ensuring consistency and accuracy across all samples.

The sodium (Na) concentration was performed using a flame emission photometer, a technique that measures the intensity of light emitted when sodium atoms are excited in a flame. This method is highly sensitive and specific for sodium, allowing precise quantification even at low concentrations.

For the analysis of potassium (K), magnesium (Mg), manganese (Mn), calcium (Ca), and iron (Fe), an atomic absorption spectrophotometer (AAS), specifically the PerkinElmer PinAAcle 500 model, was employed. The AAS technique involves light absorption at specific wavelengths corresponding to each metal, with the intensity of absorbed light directly proportional to the concentration of the element in the sample. Method validation parameters and detection limits were in accordance with the descriptions of Bulgaru et al. [30]. This instrument provides high sensitivity and accuracy, making it a reliable choice for essential minerals quantification in complex matrices. This methodological approach ensures accurate, reproducible, and reliable mineral content measurements, providing critical scientific data analysis and interpretation.

### 2.6. Granulosity

For granulosity (Gr) determination, the analyzed material (100 ± 0.01 g) was weighed and placed in the topmost sieve of successively decreasing apertures. The sieve aperture sizes were 118 µm, 132 µm, 150 µm, 180 µm, 212 µm, and the pan. The empty weight of each sieve was recorded before the sieve analysis began. The sieve was shaken for 6 min, after which the mass of the sample retained on each sieve was recorded (SR 90:1988).

### 2.7. Water-Holding Capacity

The water-holding capacity (WHC) of analyzed raw materials was determined using methods modified by Heywood et al. [31] and Lin et al. [32]. Briefly, 15 g of dry sample and 285 mL of distilled water were introduced in a 500 mL centrifuge bottle, and the mixture was vortexed for 10 min. It was then allowed to rest at room temperature for 30 min. This step enables the analyzed raw material particles to fully absorb water, often involving a rest period to ensure the material achieves equilibrium with the absorbed moisture. Afterward, the samples were centrifuged at 5000 rpm for 30 min. The supernatant was measured, and *WHC* was expressed as a g of water/g of analyzed raw material according to [33] using the following Formula (3):

(3) $$WHC=\frac{{(G}_{2}-G_{0})-G_{m}\;}{G_{m}\;}$$

where

*G*_0_—mass of dry bottle, g;
*G*_2_—mass of bottle after decanting, g;
*G_m_*—total analyzed material mass, g.

### 2.8. Color Parameters

The color of the flour samples was analyzed using a Chroma Meter CR-400 (Konica Minolta, Osaka, Japan) with D65 as a standard illuminant. Before performing the experiment, the colorimeter was calibrated with a white plate, a process necessary to standardize the obtained results. The CIELab color scale allowed the obtaining of the values of lightness (L*), red–green parameter (a*), yellow–blue parameter (b*), chromaticity (C*), and yellowing index (YI) [34]. The color parameters analysis was performed in 3 replicates at a temperature of 21 ± 1 °C.

### 2.9. Antioxidant Activity

The antioxidant activity (AA) of plant-based materials was determined for the hydroalcoholic extracts obtained as follows: 1.0 g of sample was combined with 30 mL of 70% aqueous ethyl alcohol solution (1:30, *m*/*v*), kept for 24 h in the refrigerator, then subjected to ultrasound-assisted extraction (ISOLAB installation, Laborgeräte GmbH, Eschau, Germany) at a frequency of 37 kHz and a temperature of 45 ± 1 °C for 30 min. The extracts were centrifuged at 12,000 rpm for 25 min, and the supernatant was collected, filtered through filter paper, and stored in the refrigerator until analysis.

The Trolox equivalent antioxidant capacity (TEAC) assay by reaction with the free radical DPPH•, as described in the literature by Paulpriya et al. [35], was applied. The results were expressed in mg Trolox equivalent per gram of sample, dry weight (mg TE/g DW from calibration curve 0–500 µmol/L, (R^2^ = 0.9992) with Trolox.

The ability to capture ABTS•+ free radical cations in the TEAC assay was determined according to the method described in the literature by Arnao et al. [36]. The results were expressed as mg TE/g DW.

### 2.10. Antioxidant Activity In Vitro Digestion Model

In vitro digestion of meat analog samples was performed according to the INFOGEST 2.0 protocol (gastric phase—incubate while mixing for 2 h, at 37 °C, pH 3.0, followed by intestinal phase—for 2 h, at 37 °C, pH 7.0) [37]. The samples for analysis are collected at the end of the digestion process. Samples from in vitro gastrointestinal digests were cooled to 5 °C and centrifuged at 17,500 rpm for 10 min to allow removal of insoluble material. For further analysis, the supernatants were withdrawn and frozen.

The AA of the samples after in vitro gastrointestinal digestion was determined by the DPPH and ABTS methods (2.9). The results were expressed as mg TE/g DW of the sample [38].

### 2.11. Statistical Analysis

The measurements in this study were performed in triplicate and are presented as mean values and standard error of the mean. Calculations were performed using Microsoft Office Excel 2007 (Microsoft, Redmond, WA, USA). Statgraphics, Centurion XVI 16.1.17 (Statgraphics Technologies, Inc., The Plains, VA, USA) program was used for one-way analysis of variance (ANOVA) according to Tukey’s test at a significance level of *p* < 0.05. The Pearson correlation and principal component analysis (PCA) were computed using the Scikit-learn Python library [39].

## 3. Results and Discussion

### 3.1. Physicochemical Quality Indicators

The transition from animal proteins to plant proteins can be achieved gradually, smoothly, and orderly by using protein-rich sources, isolates, or protein concentrates from plant sources that can form a texture and flavor similar to animal proteins to completely replace meat in a meal [40]. The meat analog manufacturing industry can include a multitude of protein-rich raw materials, including legume isolates and concentrates (soy or peas), legume or cereal flours (cereals rich in gluten and starch), and, of course, oil press cakes of different vegetable sources (nuts, seeds) [17]. For these reasons, investigating the quality of plant protein sources is essential in planning the production of meat analogs. The physicochemical indicators of analyzed raw materials are presented in Table 1.

The DM content of the analyzed samples was mainly represented by the protein and carbohydrate content. The highest content was in HOC and PPI.

Based on their chemical composition, samples were an important source of proteins, with the maximum values being presented for PPI—76% and SPI and SPC—80.50% and 52.80%, respectively, followed by HOC—34.98% and CPF—22.05%.

The analyzed samples did not exhibit high FC. The results for fat content vary within the limits of 8.13% and 0.92%. The highest amounts of fat were identified for PPI (8.13%) samples, followed by CPF (5.25), COH (3.12%), and SPC (2.21%). The minimum amount of fat was recorded for the sample SPA (0.92%). Plant-based ingredients are naturally lower in fat and cholesterol than those of animal origin. It is also manifested by having a higher content of polyunsaturated fatty acids, which can satisfy about 60% of an adult’s needs [41].

The highest AC of all the samples analyzed was identified in HOC—6.57%. The AC in the PPI, SPI, and SPC was similar, with no large gaps between the values.

The SY was identified only in CPF. Starch was not identified in the other samples. According to publications by other authors, sucrose (3.97%) prevails among the carbohydrates contained in HOC, followed by glucose (0.8%), fructose (0.57%), and xylose (0.34%) [42].

Similar results for SY, PC, and AC in chickpeas were also obtained by Xiao et al. [43], showing that the analyzed varieties of chickpeas have a SY ranging from 27.15 ± 1.19 to 36.22 ± 0.55 g/100 g, PC 19.79 ± 2.89 to 23.38 ± 0.30 g/100 g, and AC ranged between 2.59 ± 0.05 and 2.69 ± 0.03 g/100 g. Similar data on the chemical composition of hazelnuts were obtained by Pycia et al. [44] and Goksu et al. [45], who showed in their work that hazelnut meals could contain up to 40% protein.

The CFC of CPF and HOC are practically similar in samples of PPI, SPI, and SPC; the CFC is small. This component will contribute to highlighting the technological properties of the end product. Van der Sman et al. [46] mentioned that to obtain good textural properties for meat analogs, the presence of both soluble and insoluble dietary fibers is definitive. They differ in their WHC, with insoluble fibers having a lower WHC than the protein phase and soluble fibers having a higher WHC than the protein phase. The difference in WHC influences the distribution of water and, respectively, the rheological properties of the finished product, where the protein phase must remain continuous to form a fiber structure. 

Slight differences between WHC values obtained by different authors could be caused by the different de-oiling processes of the hazelnut flour or the type of hazelnuts used in the studies. The content of protein and dietary fiber, which have many functional groups that tend to interact with water molecules, also greatly affect the results for that indicator [47]. For CPF, the WAC is relatively high (3.88 g water/g flour). According to Badia-Olmos et al. [48], this parameter is at around 2.14–2.21 g water/g flour. This makes CPF effective in absorbing water, contributing to its good binding and textural properties in various food formulations. There are less direct data on HOC WHC compared to other plant-based flours. However, oil cakes typically have high oil-binding capacities, which can impact their water retention due to the fats they contain. HOC may absorb some water, but their primary focus is on retaining oils, which could affect the texture and moisture retention of baked goods and other products. Its lower WHC, compared to legume-based ingredients, is due to the dominance of fat residues and lower protein and fiber levels. Its lower WHC, compared to legume-based ingredients, is due to the dominance of fat residues and lower protein and fiber levels. In contrast to SPC or PPI, HOC has significantly lower hydration due to the limited availability of hydrophilic amino acids and polar groups. WHC of PPI depends on the degree of protein denaturation and the extraction process. High WHC is attributed to the exposure of polar amino acid residues during isolation, which promotes water binding. Compared to CPF, PPI holds considerably more water due to its purity and PC, making it a preferred ingredient in applications requiring moisture retention. Studies by Farhi et al. [49] indicate that PPI often rivals SPI in WHC. SPC shows a WHC of 4.97 g water/g protein, positioning it slightly higher than PPI. The high WHC of SPC is due to its higher degree of protein aggregation and denaturation, which increases water-binding sites. Compared with SPI, SPC typically holds less water because of the presence of some non-protein components (e.g., carbohydrates), which reduce overall hydration capacity. SPI exhibits the highest WHC among the analyzed materials, ranging from 5.54 g water/g protein. Its high WHC is due to the removal of non-protein components, leading to a high concentration of hydrophilic amino acids and a more extensive protein network. In comparison with PPI and SPC, SPI demonstrates superior WHC, particularly in gelation and emulsification applications. Research by Wang et al. [50] shows that SPI’s WHC is influenced by pH and ionic strength, with optimal hydration observed at neutral pH. Compared with PPI, CPF generally exhibits a lower WHC because PPI is highly refined and has superior hydrophilic properties. However, CPF’s WHC is more comparable to SPC.

The particle size distribution depends on the granule structure, the degree of processing, and the chemical composition of the analyzed material [51]. Higher values of Gr indicate a higher content of finer particles in the material and may explain the higher WHC. The finer the particle size of the analyzed raw material, the higher the rate and extent of water absorption. In addition, the particle size distribution of the studied samples has a great influence on their functional properties, such as swelling and water-binding capacity, which may significantly affect the quality of the resulting analogs [52]. Gr values of analyzed materials range from 134 µm SPC to 205 µm HOC. Smaller particle-size materials (e.g., SPC, SPI) tend to have higher WHC values. Larger particle-size materials (e.g., HOC) exhibit lower WHC. There appears to be an inverse correlation between particle size (Gr) and WHC: smaller particles provide a larger surface area relative to their volume, enhancing water absorption. Materials with larger particle sizes show reduced WHC: SPI (Gr—135 µm, WHC—5.54 g water/g) has high WHC and small particles. HOC (Gr—205 µm, WHC—2.17 g water/g) has low WHC and large particles. SPI, SPC, and PPI show consistently high WHC compared to CPF and HOC, likely due to their hydrophilic amino acid residues. CPF exhibits moderate WHC, possibly due to its starch content.

The color attributes of analyzed plant-based materials were evaluated using the CIELab color space parameters: lightness (L*), red–green parameters (a*), yellow–blue parameters (b*), chromaticity (C*), and yellowness index (YI). These parameters provide insights into the visual properties, which are critical for food applications, influencing consumer perception and product formulation. The L* value represents the lightness of the sample, with higher values indicating a lighter color. CPF exhibited the highest L* value (90.59), indicating its light color, and SPC also showed a high lightness value of 88.87. HOC had the lowest L* value (51.62), signifying a darker appearance, likely due to the oil extraction process and high residual phenolic content. The a* value describes the red–green color axis, where positive values indicate redness and negative values indicate greenness. HOC had the highest positive a* value (6.97), showing a distinct reddish hue. In contrast, CPF and SPC showed slightly negative a* values (−1.00 and −1.06, respectively), reflecting a mild greenish tint. The b* value measures the yellow–blue axis, with higher values indicating more yellowness. HOC and PPI showed the highest b* values (22.45 and 21.55, respectively), indicating strong yellow tones. SPC exhibited lower b* values of 14.98, indicating less pronounced yellow hues. C* represents the saturation or intensity of the color, calculated from a* and b* values. HOC exhibited the highest C* (23.51), indicating the most saturated color among the samples. CPF and PPI also had high C* values (19.67 and 21.71), contributing to their vibrant appearance. The yellowness index quantifies the degree of yellowness, which is often used to assess flour and protein concentrates. HOC displayed the highest yellowness index (62.13), aligning with its strong yellow and red hues. CPF and SPI also had relatively high yellowness indices (30.97 and 31.69, respectively). SPC showed lower YI values (24.08), indicating a paler, less yellow appearance. The observed color variations among the samples reflect their compositional differences, which affect their potential applications in food products. CPF has high lighter and moderate yellowness and is suitable for light-colored formulations. HOC, with its dark and intense color, may be used as a functional ingredient where color intensity is desirable. PPI, SPI, and SPC showed intermediate color attributes, making them versatile for applications where color uniformity and mild yellow hues are beneficial. The differences in yellowness among these protein products can influence consumer acceptance of plant-based dairy alternatives, meat analogs, and nutritional powders.

Plant-based sources rich in proteins, due to their functional properties, have the potential to present high antioxidant properties. The AA in hydroalcoholic extracts of vegetable protein materials (1 g of sample in 30 mL of 70% ethanol aqueous solution) was determined by the DPPH and ABTS methods (the quenching capacity of the free radical DPPH (2,2-diphenyl-1-picrylhydrazyl-hydrate) and the free radical cation ABTS (2,2-azinobis-(3-ethylbenzothiazoline-6-sulfonate), Table 1. All samples showed AA, which, according to the bibliographic sources listed below, correlates with the content of polyphenols, vitamins, amino acids, and other biologically active compounds contained in the analyzed samples mentioned in this study.

The highest AA values were recorded for HOC because it contains both the shell and the kernel. Thus, in the DPPH method, HOC extracts showed values of 2.09 mg TE/g DW, and in the ABTS assay, 4.07 mg TE/g DW (Table 1). Ozdemir et al. [47] determined HOC values in the DPPH assay of 2.90 μmol TE/g for aqueous extracts and, in the ABTS assay, 19.94 μg TE/g, which are lower compared to those recorded in this work.

In research conducted by Jake et al. [53], hazelnut skin extracts demonstrated a very significant DPPH radical inhibition capacity, ranging between 820 and 1190 μmol TE/g, and the ABTS test gave values between 124 and 180 μmol TE/g.

According to bibliographic sources, HOC is a rich source of polyphenolic antioxidants, including quinic acid, quercetin-3-O-rhamnoside, (+)-catechin, catechol, glansreginin A, glansreginin B, syringic acid hexose esters, procyanidin dimer, trimer, and tetramers [54]. The antioxidant properties of hazelnut cake are due not only to the polyphenols but also to the volatile compounds, vitamins, and amino acids it contains [47].

The research of samples’ antioxidant properties (Table 1) showed that SPC has a more pronounced AA of 1.36 and 3.76 mg TE/g DW in DPPH and ABTS assays compared to SPI of 0.45 and 0.90 mg TE/g DW, respectively. These data indicate that the processing of soy flour and the obtaining of protein isolates decreases the antioxidant activity of the resulting products. Bibliographic data show that AA determined in hydromethanolic extracts for soy flour by the ABTS test is almost two times higher, and by the DPPH test, it is almost three times higher compared to that of soy concentrate [55].

Lim et al. [56] established that the AA of soybeans depends strongly on their color, being higher in the case of black beans (1.07 mg vitamin C equivalent (VCE)/g DW in DPPH assay and 4.43 mg VCE/g DW in ABTS test for 80% hydro-methanolic extracts) and brown beans (0.85 and 3.86 mg VCE/g DW in DPPH and ABTS assays, respectively). Light-colored beans exhibited AA almost three times lower in DPPH and approximately six times lower in ABTS assay. The results confirm that soy flour and derivatives are important sources of protein, minerals, antioxidants, and other phytochemicals, important ingredients for the production of functional foods.

For PPI extracts, values of 0.99 and 2.70 mg TE/g DW were recorded in the DPPH and ABTS tests, which are slightly higher compared to the bibliographic data obtained for hydromethanolic extracts (20%) from pea flour [57].

Amarowicz et al. [58] determined, in 80% aqueous acetone extracts of pea seeds (*Pisum sativum*), a higher antioxidant activity of 0.30 μmol Trolox/mg. Other research has shown, in 50% aqueous acetone extracts of pea seeds, DPPH values of 1.21–2.09 μmol Trolox/g [59].

Nilsson et al. [60] determined by the ferric ion reducing antioxidant power (FRAP) method the total antioxidant activity of aqueous (0.20 to 1.29 μmol/g) and acetone (from 0.05 to 0.46 μmol/g) extracts of green peas after blanching and freezing. Research showed that pea seeds are not only an accessible source of antioxidants and protein but also contain soluble and insoluble fiber, unsaturated fatty acids, minerals, and carbohydrates, and the advantage of pea flour and protein isolates is their longer shelf life [59].

In CPF extracts, the scavenging capacity of DPPH radical and ABTS cation radical was also estimated, with values of 0.61 and 0.92 mg TE/g DW, respectively. These data are very close to the values obtained by Constantini et al. [57] for the hydroethanolic extract (20%) of CPF, which did not exceed 0.68 mg TE/g DW in both tests. Research in the field has shown that purified peptide fractions from the chickpea albumin hydrolysate had ABTS scavenging activity with values ranging between 0.550 and 0.967 mmol TE/L of aqueous extract (0.5 mg/mL) [61]. The antioxidant activity determined in dry extracts of biologically active compounds from six chickpea species had maximum IC50 values of 1.0 mg/mL in the DPPH assay and 2.3 mg/mL of extract in the ABTS assay [62].

Research has confirmed that CPF has antioxidant properties and is an accessible source of protein with a balanced amino acid composition, and bibliographic sources also mention that chickpeas have a low content of antinutrients [63].

The analyzed samples presented a broad profile of amino acids, including essential ones. The results are presented in Table 2.

In the samples of CPF, as well as in HOC, 18 different amino acids were identified, and in the SPI, SPC, and PPI samples, 17 types were identified; the amino acid γ-aminobutyric acid was not identified. The majority of amino acids are glutamic acid, leucine, arginine, phenylalanine, serine, valine, alanine, and tyrosine. Chickpea proteins are a good and balanced source of essential amino acids with high bioavailability [64]. Several sources have shown that the protein digestibility of chickpeas is between 48 and 89% [65,66]. Obtaining results indicated that glutamic acid is the most abundant non-essential amino acid across all samples, especially in PPI (80.24 g/kg) and SPI (78.58 g/kg). In the HOC, glutamic acid, 37.62%, was determined to be in the highest amount, followed by arginine (18.43%) and aspartame (15.74%). Leucine and valine showed the highest values among the essential amino acids. Proline and alanine are also relatively abundant in SPI and PPI compared to whole food sources like CPF and HOC. The amino acid composition of HOC is consistent with the values for amino acid content reported by other authors [47]. The same trend was observed in the case of SPI and SPC, where the majority of amino acids were presented by glutamic acid. Analyses of essential amino acids indicated that PPI and SPI are significantly richer in essential amino acids compared to other samples, indicating their potential as excellent sources for complete proteins. Leucine is notably high in PPI (66.71 g/kg) and SPI (43.42 g/kg). Lysine is exceptionally high in PPI (45.81 g/kg), an important amino acid often limiting in plant-based diets. Immunoactive amino acids include glutamine, arginine, and others that support immune function. PPI and SPI show the highest values, making them promising for functional food applications. Amino acids for energy metabolism, particularly glycogenic amino acids (converted to glucose) and ketogenic amino acids (converted to ketone bodies), are abundant in isolates, which could support energy production during intense activity or fasting. Sulfur-containing amino acids, especially cysteine and methionine, essential for antioxidant functions and metabolic processes, are highest in SPI (12.32 g/kg). Total amino acids associated with nitrogen metabolism were highest in PPI and SPI, reflecting their high protein quality and completeness.

The studied raw materials are also an important source of mineral salts, essential for the consumer’s health. The results of the mineral salts profile analysis are presented in Table 3.

The carried-out analysis of the mineral salt profile showed the highest amounts for the content of K, Mg, and Ca for all samples. In CPF, in descending order, there were K > Mg > Ca > Fe > Mn > Na. For HOC, K > Mg > Ca > Mn > Fe > Na. In these materials, the Na content is in minimal quantities. On the other hand, in the samples of SPI and PPI, the mineral salts profile is presented mainly by the Na content, followed by K, Ca, and Mg. In SPC, in major quantities were K, followed by Ca, Fe, Na, Mn, and Mg. Combining these ingredients in the recipe for manufacturing meat analogs would allow for obtaining a final product with a balanced mineral salt intake.

Varieties, location, soil composition, fertilizer use, and irrigation affect the chemical composition of the studied raw material types, including the content of mineral salts. In this context, it is more difficult to compare the data obtained with those obtained by other authors. Cabreraet al. [67] showed that chickpeas are notable for their impressive content of essential minerals, such as Fe (5.0 mg/100 g), Zn (4.1 mg/100 g), Mg (138 mg/100 g), and Ca (160 mg/100 g). Fatemehet al. [68] also showed a major content of K in studied hazelnut varieties, followed by P and Mg.

### 3.2. Results of Antioxidant Activity In Vitro Digestion Model

**Figure 2:**
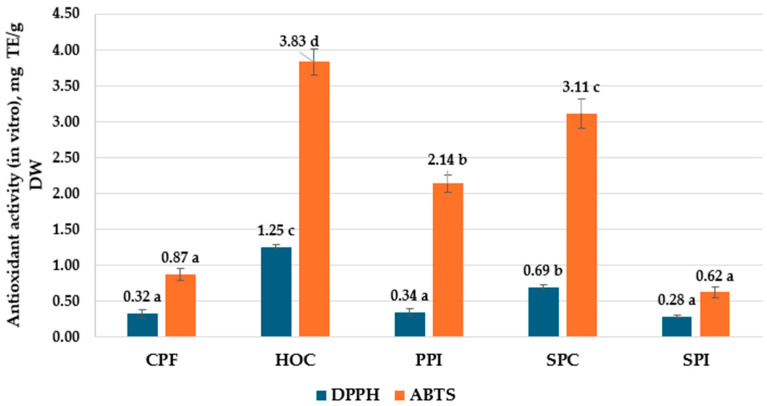
Antioxidant activity (DPPH, ABTS) in vitro of analyzed samples (CPF—chickpea flour, HOC—hazelnut oil cake, PPI—pea protein isolate, SPC—soy protein concentrate, SPI—soy protein isolate). Different letters (^a–d^) designate statistically different results (*p* ≤ 0.05).

Another objective of this research was to study how the in vitro simulated digestion (INFOGEST protocol) influences the antioxidant activity of biologically active compounds from vegetable protein materials. In the samples taken after the gastric and intestinal digestion simulation, the Trolox equivalent antioxidant capacity was determined by the DPPH and ABTS methods (Figure 2). The research carried out in this study showed that the samples obtained after intestinal digestion have a fairly significant AA, with maximum values for HOC (1.25 mg TE/g DW) and minimum for CPF (0.32 mg TE/g DW) recorded in the DPPH test. The values determined in the ABTS method are comparatively higher, with maximums of 3.83 mg TE/G DW (HOC) and 0.62 mg TE/g DW (SPI).

For DPPH antioxidant activity, there is no significant difference among CPF, PPI, and SPI. These samples exhibit the lowest antioxidant activities, with values ranging from 0.28 to 0.34 mg TE/g DW. In contrast, SPC shows moderately higher activity at 0.69 mg TE/g DW and is labeled with the letter “b”, indicating a significant difference from the lower-performing samples. HOC demonstrates the highest antioxidant activity at 1.25 mg TE/g DW, which is statistically distinct from all other samples.

For ABTS antioxidant activity, CPI and SPI have the lowest values, measured at 0.62 and 0.87 mg TE/g DW, respectively, and categorized as group “a”. PPI demonstrates higher antioxidant activity at 2.14 mg TE/g DW, showing a statistically significant difference compared to the lower-performing samples. SPC and HOC have the highest antioxidant activities, measured at 3.11 and 3.83 mg TE/g DW, respectively.

Researchers have demonstrated that many phenolic compounds are lost along the gastrointestinal tract and, as a result, the AA of samples after digestion decreases [69]. Probably, the loss of polyphenols explains the lower values of AA in the DPPH assay (Figure 2); this free radical is quenched in particular by polyphenols through the effects of electron transfer, proton transfer, or hydrogen atom transfer [70,71].

The high values of AA in the ABTS test are due to the process of hydrolysis and denaturation of plant-based proteins in the gastrointestinal tract. Kut et al. [72] showed that protein denaturation and hydrolysis during digestion increased AA in the resulting hydrolysates due to the release of amino acids responsible for AA, such as tyrosine, tryptophan, cysteine, histidine, arginine, and cystine in the ABTS decolorization assay. At the same time, the hydrolysis of carbohydrates during digestion leads to the formation of compounds with antioxidant properties, such as reducing oligosaccharides and monosaccharides.

**Figure 3:**
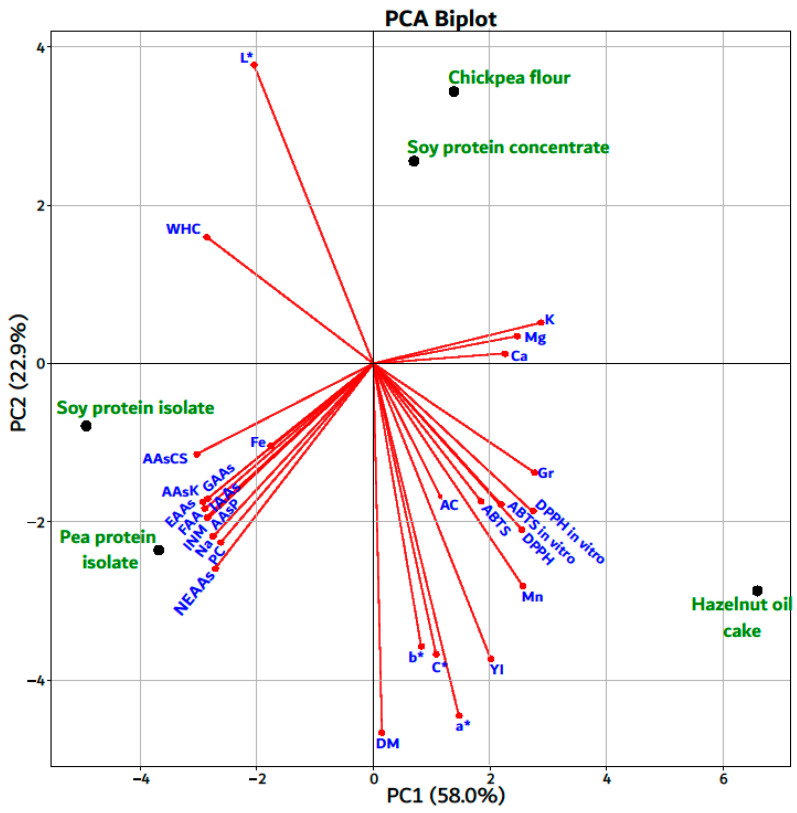
Principal component analysis; FAAs—free amino acids; INM—indicators of nitrogen metabolism; NEAAs—non-essential amino acids; EAAs—essential amino acids; IAAs—immunoactive amino acids; GAAs—glycogen amino acids; AAsK—amino acids ketogenic; AAsP—amino acids proteinogenic; AAsCS—amino acids containing S; DM—dry matter; AC—ash content; FC—fat content; PC—protein content; CFC—crude fiber content; SY—starch yield; WHC—water-holding capacity; Gr—granulosity; L*—lightness; a*—red–green parameter; b*—yellow–blue parameter; C*—chromaticity; YI—yellowing index; DPPH—2,2-diphenyl-1-picrylhydrazyl-hydrate; ABTS—2,2-azinobis-(3-ethylbenzothiazoline-6-sulfonates); Na—sodium; K—potassium; Mg—magnesium; Ca—calcium; Mn—manganese; Fe—iron.

Principal component analysis (PCA) was used to illustrate the relationship between physicochemical characteristics, amino acid compositions, minerals composition, and antioxidant activity (DPPH and ABTS) determined in the raw materials (CPF, HOC, PPI, SPC, and SPI) in terms of meat analogs manufacture (Figure 3).

The PC1 and PC2 components accounted for 58.0% and 22.9% of the total variance. Figure 3 shows that some of the analyzed parameters are close, demonstrating high significant correlations (*p* < 0.05), such as PC and compositions (m = 0.99); ∑FAAs, ∑INM, ∑AAsP (r = 0.97), of the analyzed parAAsK and sAAsCS (r = 0.93), Na (r = 0.89); K and Mg (r = 0.97), Ca (r = 0.92); C* and a* (r = 0.83), b* (r = 0.99); YI and a* (r = 0.97), b* (r = 0.81); DPPH and DPPH in vitro (r = 0.96); ABTS and ABTS in vitro (r = 0.97), Figure S1. The PC1 was closely associated with Ca, Mg, K, Gr, DPPH and ABTS (in vitro), WHC, ∑AAsCS, Fe, etc., whereas PC2 was closely associated with L*, DM, a*, b*, C*, YI, AC, Mn, etc. According to the PCA plot, the plant materials that are closest to each other are SPI and PPI, which are located on the left of the graph; CPF and SPC, which are positioned on the upper right of the PCA; and HOC, which seems to separate from the other groups of plant materials on the bottom right of the PCA, probably suggesting differences in chemical composition. Both PCA components distinguish CPF and SPC from SPI and PPI, indicating an inverse correlation between these plant materials. The first component PC1 distinguishes CPF and SPC from SPI, PPI, and HOC, while the second component PC2 distinguishes CPF, SPC, and HOC from SPI and PPI plant materials. Regarding the correlations of plant materials with the analyzed characteristics, it seems that SPI and PPI are more related to protein content, amino acid composition, and Na; ICPF and SPC with K, Mg, and Ca; HOC is related to color parameters (a*, b*, C*, YI) and AA (DPPH and ABTS).

The development of plant-based meat analogs using ingredients such as CPF, HOC, PPI, SPC, and SPI involves various aspects.

CPF, despite its high dry matter content (91.60%), has lower protein content (22.05%) and moderate water retention capacity (3.88 g/g). This indicates limited functionality in mimicking meat texture and requires blending with higher protein sources, increasing formulation complexity. Due to its high starch content (35.15%), CPF is best suited for extrusion and allows for fibrous textures. CPF has great potential for cost-effective incorporation as a binder or filler in meat analog formulations.

HOC is characterized by relatively high antioxidant capacity (6.57%), high aromatic amino acids (tyrosine and phenylalanine), and moderate protein content (34.98%). HOC has environmental advantages due to its origin as a by-product of oil production, promising as a functional ingredient in formulations aimed at oxidative stability. The lower essential amino acid profile (85.08 g/kg) of HOC may limit its independent use in high-protein products. Also, although it has the advantage of being environmentally friendly, its availability is limited geographically, potentially affecting cost stability.

CPF and HOC offer cost reduction and sustainability but require optimization to match the protein density and sensory appeal of soy- and pea-based ingredients.

PPI is ideal for manufacturing high moisture meat analog by extrusion due to its solubility and gel-forming properties. It is characterized by high protein content (76.00%) and high functional properties, including the highest amino acid composition for lysine (45.81 g/kg) and leucine (66.71 g/kg). Despite higher production costs associated with specialized extraction processes, its market demand and scalability support its feasibility.

SPC is characterized by a balanced protein content (52.80%), moderate moisture retention (4.97 g/g), and a strong composition of essential amino acids, particularly lysine (25.81 g/kg). SPC could improve freeze–thaw stability of product.

SPI, with the highest protein content (80.50%) and water-holding capacity (5.54 g/g), provides optimal texture and nutrition but may require formulation adjustments to manage its foaming and emulsifying properties. SPI outperforms other analogs in essential amino acid content (251.6 g/kg) and water-holding capacity (5.54 g/g), making it a versatile ingredient for meat analogs. Despite the range of benefits of SPC and SPI, there are concerns about the energy intensity of soya cultivation, environmental impacts, and allergenicity, which may affect consumer preferences. Incorporating these ingredients into meat analog formulations requires balancing nutritional benefits, sensory attributes, and production economics. While soy-derived proteins lead the way in terms of functionality and economics, pea protein isolate provides a viable alternative for allergen-free markets.

The use of CPF, HOC, PPI, SPC, and SPI as meat analogs offers significant sustainability benefits by reducing the environmental impact associated with traditional meat production. Chickpea meal, derived from a highly efficient moisture- and nitrogen-fixing crop, contributes to soil health while requiring relatively low agricultural inputs. Hazelnut cake, a by-product of oil extraction, exemplifies the principles of the circular economy by converting waste into a functional protein ingredient, thereby minimizing food waste streams. Pea protein isolate, produced from a legume crop with low greenhouse gas emissions and water consumption, supports sustainable agricultural practices and contributes to crop rotation strategies that improve biodiversity. Soy protein concentrate and soy protein isolate derived from high-yielding soya beans are efficient sources of protein with a lower carbon footprint than animal protein, but their sustainability can be enhanced through responsible sourcing that reduces deforestation and improves land use efficiency. The use of these plant-based ingredients in the production of meat analogs not only reduces dependence on resource-intensive livestock production but also supports global food security by offering nutrient-rich alternatives with lower environmental impact, which is in line with sustainable development and climate change mitigation goals.

Overall, the feasibility of using the reviewed ingredients as meat analogs depends on targeted product development, resource-efficient processing technologies, and alignment with evolving consumer trends that prioritize health, sustainability, and affordability.

Sustainability considerations favor the use of by-product-based materials such as HOC, while PPI, SPC, and SPI require strategies to offset high production costs and environmental impacts through circular production models or renewable energy integration.

## 4. Conclusions

Investigation of the plant-based raw material quality indices (chickpea flour (CPF), peanut oil cake (HOC), pea protein isolate (PPI), soy protein concentrate (SPC), soy protein isolate (SPI)) with potential for use in the manufacture of meat analogs demonstrated that they are suitable for use in the manufacture of this category of food products. The maximum values for protein content were identified in IPP—76% and SPI and SPC—80.50% and 52.80%, respectively, followed by HOC—34.98% and CPF—22.05%. The crude fiber content (CFC) was the highest in SPI—4.03%, followed by CPF and HOC, 1.96 and 1.98%, respectively. The particle sizes range from 134 µm (SPC) to 205 µm (HOC). Materials with smaller particle sizes have values of higher water-holding capacity (WHC). Color variations between samples were determined, reflecting their compositional differences, and should be taken into account for potential applications in food products. The analysis of the amino acid composition demonstrated the presence of 18 (CPF, HOC) and 17 (PPI, SPC, SPI) amino acids, with glutamic acid, leucine, arginine, phenylalanine, serine, valine, alanine, and tyrosine being predominant. The individual composition and total amino acids associated with nitrogen metabolism reflect the high quality and completeness of the analyzed vegetable proteins. The analysis of the mineral composition of the raw materials demonstrated that they also present an important source of K, Mg, and Ca, essential for consumer health. The antioxidant activity of biologically active compounds from vegetable protein materials was analyzed after simulating gastric and intestinal digestion by the DPPH and ABTS methods. SPC and HOC exhibited the highest antioxidant activities: 3.11 and 3.83 mg TE/g DW, respectively. Principal component analysis (PCA), used to illustrate the relationship between physicochemical characteristics, amino acid composition, mineral composition, and antioxidant activity (DPPH and ABTS), allowed the characterization of the specificity of plant raw materials (CPF, HOC, PPI, SPC, and SPI) in terms of use as meat analogs in different formulations. The use of CPF, HOC, PPI, SPC, and SPI as meat analogs offers significant sustainability benefits by reducing the environmental impact associated with traditional meat production.

## 5. Future Outlook

Health-conscious consumers are more likely to choose plant-based protein products, especially if they are high in fiber and contain less saturated fat. Plant-based protein sources are potential raw materials in the manufacture of meat analogs, taking into account sustainability issues and the use of emerging processing techniques, as well as providing dietary fiber, active ingredients with high antioxidant potential that increase the functionality of the final product. These products can also offer economic and environmental advantages.

To ensure the ever-increasing demand for meat analog products, constant investigations are needed to improve taste and quality. Advances in food technology allow the creation of products that sensory approach the taste, texture, and appearance of traditional meat.

Another research direction would be to diversify the varieties of plant-based meat analog products. These should take into account traditional products and the eating habits of the population. The presence of a wide range of plant-based meat analog products in grocery stores and restaurants will facilitate consumer acceptance of this type of product.

## Figures and Tables

**Table 1:** Physicochemical indices, color parameters, and antioxidant activity of analyzed plant-based materials.

Indices	Analyzed Plant-Based Materials				
	CPF	HOC	PPI	SPC	SPI
DM, %	91.60 ± 0.11 ^b^	93.20 ± 0.09 ^d^	93.00 ± 0.07 ^d^	90.80 ± 0.15 ^a^	92.40 ± 0.13 ^c^
AC, %	2.99 ± 0.03 ^a^	6.57 ± 0.09 ^d^	4.10 ± 0.03 ^b^	6.44 ± 0.05 ^d^	5.33 ± 0.07 ^c^
FC, %	5.25 ± 0.03 ^d^	3.12 ± 0.06 ^c^	8.13 ± 0.05 ^e^	2.21 ± 0.04 ^b^	0.92 ± 0.02 ^a^
PC, %	22.05 ± 0.31 ^a^	34.98 ± 0.26 ^b^	76.00 ± 0.42 ^d^	52.80 ± 0.34 ^c^	80.50 ± 0.39 ^d^
CFC, %	1.96 ± 0.0 ^b^	1.98 ± 0.0 ^b^	0.19 ± 0.01 ^a^	0.20 ± 0.01 ^a^	4.03 ± 0.04 ^c^
SY, %	35.15 ± 0.16	-	-	-	-
WHC, g water/g material	3.88 ± 0.07 ^b^	2.17 ± 0.05 ^a^	4.62 ± 0.03 ^c^	4.97 ± 0.06 ^d^	5.54 ± 0.04 ^d^
Gr, µm	170 ± 4 ^b^	205 ± 4 ^c^	140 ± 1 ^a^	134 ± 4 ^a^	135 ± 3 ^a^
L*	90.59 ± 1.15 ^c^	51.62 ± 0.76 ^a^	81.12 ± 0.56 ^b^	88.87 ± 1.04 ^c^	83.44 ± 0.53 ^b^
a*	−1.00 ± 0.03 ^a^	6.97 ± 0.09 ^d^	2.65 ± 0.06 ^c^	−1.06 ± 0.04 ^a^	1.20 ± 0.05 ^b^
b*	19.64 ± 0.07 ^b^	22.45 ± 0.10 ^c^	21.55 ± 0.09 ^c^	14.98 ± 0.16 ^a^	18.51 ± 0.13 ^b^
C*	19.67 ± 0.04 ^b^	23.51 ± 0.09 ^d^	21.71 ± 0.06 ^c^	15.02 ± 0.10 ^a^	18.55 ± 0.07 ^b^
YI	30.97 ± 0.11 ^b^	62.13 ± 0.57 ^d^	37.95 ± 0.31 ^c^	24.08 ± 0.24 ^a^	31.69 ± 0.51 ^b^
AA (DPPH), mg TE/g DW	0.61 ± 0.04 ^a^	2.09 ± 0.03 ^d^	0.99 ± 0.01 ^b^	1.36 ± 0.01 ^c^	0.45 ± 0.05 ^a^
AA (ABTS), mg TE/g DW	0.92 ± 0.03 ^a^	4.07 ± 0.10 ^d^	2.7 ± 0.08 ^b^	3.76 ± 0.05 ^c^	0.90 ± 0.04 ^a^

The antioxidant activity of ABTS and DPPH was determined for hydroethanolic extracts of raw materials. DM—dry matter; AC—ash content; FC—fat content; PC—protein content; CFC—crude fiber content; SY—starch yield; WHC—water-holding capacity; Gr—granulosity; L*—lightness; a*—red–green parameter; b*—yellow–blue parameter; C*—chromaticity; YI—yellowing index; AA—antioxidant activity; DPPH—2,2-diphenyl-1-picrylhydrazyl-hydrate; ABTS—2,2-azinobis-(3-ethylbenzothiazoline-6-sulfonates); TE—Trolox equivalent. The results are mean ± standard deviation. Different letters (^a–e^) designate statistically different results (*p* ≤ 0.05). CPF—chickpea flour, HOC—hazelnut oil cake, PPI—pea protein isolate, SPC—soy protein concentrate, SPI—soy protein isolate.

**Table 2:** Amino acid compositions of analyzed plant-based materials in terms of meat analog manufacture.

Amino Acids, g/kg	Analyzed Plant-Based Material				
	CPF	HOC	PPI	SPC	SPI
Aspartame	20.13 ± 1.03 ^a^	15.74 ± 0.97 ^a^	62.43 ± 1.32 ^c^	40.69 ± 1.28 ^b^	63.16 ± 2.13 ^c^
Threonine	7.91 ± 0.17 ^a^	7.58 ± 0.32 ^a^	18.17 ± 0.25 ^c^	13.70 ± 0.41 ^b^	21.26 ± 0.18 ^d^
Serin	7.69 ± 0.21 ^a^	12.33 ± 0.15 ^b^	26.42 ± 0.29 ^d^	20.30 ± 0.11 ^c^	30.19 ± 0.45 ^e^
Glutamic	25.72 ± 0.34 ^a^	37.62 ± 1.11 ^b^	80.24 ± 1.78 ^d^	46.70 ± 0.56 ^c^	78.58 ± 1.47 ^d^
Proline	10.62 ± 0.21 ^a^	15.86 ± 0.75 ^b^	28.92 ± 0.67 ^c,d^	25.02 ± 0.32 ^c^	39.55 ± 0.89 ^e^
Glycine	7.56 ± 0.42 ^a^	10.72 ± 0.64 ^b^	23.80 ± 1.02 ^e^	14.77 ± 0.43 ^c^	20.75 ± 0.47 ^d^
Alanine	8.79 ± 0.15 ^b^	4.71 ± 0.42 ^a^	25.44 ± 0.85 ^d^	16.17 ± 0.31 ^c^	26.21 ± 0.56 ^d^
Valin	8.35 ± 0.22 ^a^	13.30 ± 0.41 ^b^	25.51 ± 1.03 ^c^	14.95 ± 0.67 ^b^	27.32 ± 0.81 ^d^
Cysteine	1.84 ± 0.06 ^a^	1.49 ± 0.03 ^a^	4.14 ± 0.26 ^c^	2.48 ± 0.05 ^b^	6.61 ± 0.09 ^d^
Methionine	3.04 ± 0.08 ^b^	1.97 ± 0.05 ^a^	6.21 ± 0.07 ^e^	4.20 ± 0.09 ^c^	5.70 ± 0.14 ^d^
Isoleucine	14.83 ± 0.19 ^b^	9.26 ± 0.11 ^a^	24.63 ± 0.24 ^d^	16.75 ± 0.37 ^b,c^	25.99 ± 0.17 ^e^
Leucine	15.08 ± 0.26 ^a^	14.57 ± 0.32 ^a^	66.71 ± 1.08 ^d^	23.49 ± 0.56 ^b^	43.42 ± 0.98 ^c^
Tyrosine	4.77 ± 0.31 ^a^	8.36 ± 0.20 ^a^	24.52 ± 0.96 ^d^	11.02 ± 0.54 ^b^	21.39 ± 0.56 ^c,d^
Phenylalanine	11.29 ± 0.13 ^a^	9.94 ± 0.06 ^a^	27.67 ± 0.43 ^c^	17.85 ± 0.62 ^b^	35.72 ± 0.60 ^d^
*γ*-aminobutyric	0.29 ± 0.02 ^a^	0.55 ± 0.02 ^b^	nd	nd	nd
Lysine	17.02 ± 0.17 ^b^	5.03 ± 0.41 ^a^	45.81 ± 0.57 ^e^	25.81 ± 0.28 ^c^	35.91 ± 0.20 ^d^
Histidine	8.05 ± 0.08 ^b^	4.99 ± 0.12 ^a^	16.26 ± 0.19 ^c^	9.79 ± 0.06 ^b^	16.57 ± 0.34 ^c^
Arginine	16.54 ± 0.29 ^a^	18.43 ± 0.16 ^a^	58.94 ± 0.51 ^d^	25.84 ± 0.18 ^b^	39.75 ± 0.41 ^c^
Ammonia	1.89 ± 0.05 ^a^	4.87 ± 0.08 ^c^	4.94 ± 0.13 ^c^	3.21 ± 0.07 ^b^	5.97 ± 0.08 ^d^
FAAs	189.6 ± 1.3 ^a^	192.5 ± 0.9 ^a^	565.9 ± 1.4 ^c^	329.6 ± 1.6 ^b^	538.1 ± 1.1 ^c^
INM	191.5 ± 0.9 ^a^	197.3 ± 1.8 ^a^	570.8 ± 1.9 ^c^	332.8 ± 1.2 ^b^	544.1 ± 2.4 ^c^
NEAAs	87.14 ± 0.78 ^a^	106.8 ± 1.3 ^a^	275.9 ± 1.8 ^b^	117.2 ± 0.9 ^a^	286.5 ± 1.7 ^b^
EAAs	102.2 ± 0.8 ^a^	85.08 ± 0.76 ^a^	289.9 ± 1.5 ^d^	152.4 ± 1.1 ^b^	251.6 ± 1.3 ^c^
IAAs	80.74 ± 0.73 ^a^	93.33 ± 1.04 ^a^	242.4 ± 0.9 ^c^	155.0 ± 0.7 ^b^	253.4 ± 0.8 ^c^
GAAs	60.45 ± 0.21 ^a^	64.38 ± 0.19 ^a^	181.8 ± 1.1 ^c^	120.6 ± 0.8 ^b^	188.9 ± 0.6 ^c^
AAsK	62.99 ± 0.35 ^b^	47.17 ± 0.56 ^a^	189.3 ± 1.0 ^e^	94.94 ± 0.27 ^c^	162.4 ± 0.51 ^d^
AAsP	189.3 ± 1.3 ^a^	191.9 ± 1.1 ^a^	565.9 ± 2.4 ^c^	329.6 ± 1.9 ^b^	538.1 ± 1.5 ^c^
AAsCS	4.93 ± 0.07 ^b^	3.46 ± 0.11 ^a^	10.35 ± 0.25 ^d^	6.68 ± 0.18 ^c^	12.32 ± 0.11 ^e^

FAAs—free amino acids; INM—indicators of nitrogen metabolism; NEAAs—non-essential amino acids; EAAs—essential amino acids; IAAs—immunoactive amino acids; GAAs—glycogen amino acids; AAsK—amino acids ketogenic; AAsP—amino acids proteinogenic; AAsCS—amino acids containing S. The results are mean ± standard deviation. Different letters (^a–e^) designate statistically different results (*p* ≤ 0.05); nd—not detected. CPF—chickpea flour, HOC—hazelnut oil cake, PPI—pea protein isolate, SPC—soy protein concentrate, SPI—soy protein isolate.

**Table 3:** Mineral composition of analyzed plant-based materials.

Minerals, mg/kg	Analyzed Plant-Based Materials				
	CPF	HOC	PPI	SPC	SPI
Sodium (Na)	32.8 ± 1.6 ^a^	11.0 ± 0.9 ^a^	10,537 ± 23 ^b^	192.5 ± 11.6 ^a^	15,375 ± 29 ^c^
Potasium (K)	12,085 ± 15 ^b^	29,219 ± 41 ^d^	860.0 ± 12.1 ^a^	23,925 ± 37 ^c^	2262 ± 16 ^a^
Magnesium (Mg)	1405 ± 9 ^b^	3792 ± 15 ^c^	696.9 ± 8.9 ^a^	3798 ± 11 ^c^	1042 ± 19 ^a^
Calcium (Ca)	1254 ± 31 ^a^	3656 ± 17 ^b^	1230 ± 12 ^a^	4012 ± 10 ^b^	951.3 ± 6.2 ^a^
Manganese (Mn)	25.0 ± 1.2 ^a^	1320 ± 23 ^b^	30.6 ± 1.4 ^a^	45.3 ± 1.0 ^a^	17.3 ± 0.4 ^a^
Iron (Fe)	55.0 ± 0.8 ^a^	137.5 ± 3.5 ^b^	202.5 ± 5.1 ^c^	263.3 ± 3.7 ^d^	270.0 ± 2.9 ^d^

The results are mean ± standard deviation. Different letters (^a–d^) designate statistically different results (*p* ≤ 0.05). CPF—chickpea flour, HOC—hazelnut oil cake, PPI—pea protein isolate, SPC—soy protein concentrate, SPI—soy protein isolate.

## Data Availability

The original contributions presented in the study are included in the article. Further inquiries can be directed to the corresponding author.

## Supplementary Materials

The following supporting information can be downloaded at: https://www.mdpi.com/article/10.3390/foods14030483/s1, Figure S1: The correlation values between physicochemical characteristics, amino acid composition, mineral composition, and antioxidant activity (DPPH and ABTS) before and after in vitro digestion determined in the raw materials (chickpea flour, hazelnut oil cake, pea protein isolate, soy protein concentrate, and soy protein isolate).
