# Supplementary figures and images for: Characterization of Plant-Based Raw Materials Used in Meat Analog Manufacture
Source: Foods. 2025 Feb 3;14(3):483. doi: 10.3390/foods14030483 (PMC11817097; doi:10.3390/foods14030483)

# PCA Biplot

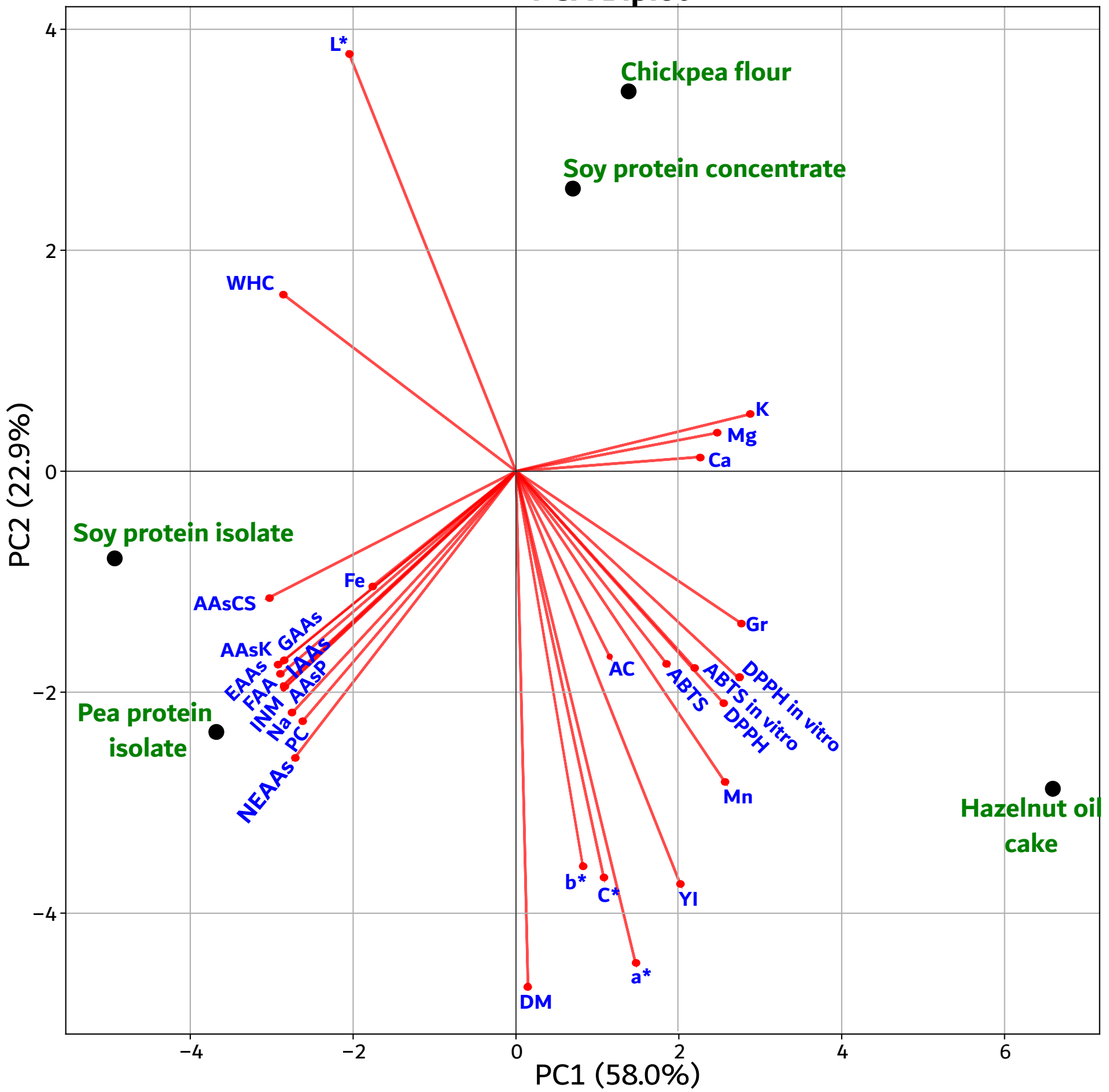

Supplement: Supplementary file 1 [file foods-14-00483-s001.zip › foods-3396738-supplementary.pdf]
